# Social learning of scientific and religious views: parents as transmission agents and their approaches against dissent

**DOI:** 10.3389/fpsyg.2026.1870472

**Published:** 2026-08-20

**Authors:** Frankie T. K. Fong, Eilidh Meighan, Inkuk Kim, Nur Amni Aqilah Mohamed Izam, Kathleen H. Corriveau

**Affiliations:** 1 School of Psychological Sciences, Victoria University of Wellington, Wellington, New Zealand; 2 School of Psychology, University of Queensland, Brisbane, QLD, Australia; 3 Department of Comparative Cultural Psychology, Max Planck Institute for Evolutionary Anthropology, Leipzig, Germany; 4 School of Psychology, Sunway University, Bandar Sunway, Malaysia; 5 Wheelock College of Education and Human Development, Boston University, Boston, MA, United States

**Keywords:** belief, cultural transmission, parents, religion, science

## Abstract

Focusing on two prominent belief frameworks – science and religion – this study examined adults’ retrospective perceptions of the role their parents played in transmitting and maintaining beliefs. We adapted a previous method (McLoughlin, 2021) and asked Australian and Malaysian adults to reflect on whether and how their current scientific and religious beliefs were influenced by their parents. Across both countries, adults indicated that their parents were influential for both science and religion. Participants generally recalled scientific influence positively irrespective of how religious they were. For religion, greater participant religiosity was associated with positive influence for Australian adults only. Approximately three-quarters of Malaysian participants identified their parents as the only source for both religious and scientific beliefs. While the same proportion of Australians indicated so for science, significantly less Australian participants identified their parents as the only source for religious beliefs. When reflecting on how their parents approached conflicting views between themselves and their parents, Australian participants generally reported that their parents employed a supportive approach across both domains. Malaysian participants often reported that their parents, though open to accepting dissenting scientific beliefs with a supportive stance, were more inclined toward a directive approach when faced with conflicts in religious views. This study affords novel insights into the universality and variation of parents’ roles in the transmission and maintenance of scientific and religious beliefs across diverse populations.

## Introduction

One distinctive feature of human culture is the presence of values and beliefs that guide how we conceptualize life events. Science and religion are two prominent domains of belief that play a vital role in humans’ explanations of the world (Ferngren, 2022; Harrison, 2006; Sivasundaram, 2010). For example, natural disasters may be understood scientifically as the result of geological or climatic processes, whereas religious perspectives may explain them as acts of divine will or responses to moral wrongdoings. Notably, many phenomena explained by science and religion – such as how germs make us sick, and that God listens to prayers – are unobservable and difficult to visualize and understand via observations. Due to this characteristic, adults and children rely predominantly on the testimony of others, rather than their own experiences, when acquiring scientific and religious beliefs (for a recent review, see Ma et al., 2024; also see Harris and Koenig, 2006). Parents play a central role in this process, acting as key transmission agents who share their beliefs with their children and encourage their adoption through social interactions and the environments they create in the home (Braswell et al., 2012; Grusec and Davidov, 2008; Kelley et al., 2021; Unser, 2025).

Existing socialization research has explored the specific ways parents scaffold children’s learning in everyday interactions, including encouraging inquiry behaviors, using guided questions and dialogic strategies, and providing explanatory talk, corrective feedback, and connections to real-world phenomena (Fender and Crowley, 2007; Setioko and Ding, 2022; Strickler-Eppard et al., 2019). Contextual factors seem to impact such transmission processes. Tenenbaum et al. (2005) found that in low-income families, mothers engaged more in scientific conversations with older compared to younger children, and did so more frequently with boys than with girls, potentially reflecting children’s increasing cognitive competence with age, the masculine-stereotyped nature of materials, or parents’ responsiveness to children’s perceived interests or preferences. These differences in scientific conversations predicted science literacy abilities and confidence in children, impacting academic pathways and exemplifying how parental knowledge transmission is fundamental for children’s development (Halim et al., 2018; Perera, 2014). More broadly, scientific attitudes and knowledge - and the processes by which they develop and interact - may vary between cultures, reflecting differences in sociocultural norms and educational experiences. Such differences are evident in beliefs about what constitutes legitimate epistemic authority, the relative emphasis on innate learning ability versus effort, and openness to scientific instruction (Allum et al., 2008; Karabenick and Moosa, 2005; Yang, 2015). Similarly, within some cultural contexts, family belief systems may also shape children’s science-related perspectives; for instance, in a United States (U.S.) report, children raised in highly religious families exhibited lower levels of science literacy and more negative attitudes toward science (McPhetres and Zuckerman, 2018).

Religious socialization tends to focus on the transmission of religious beliefs, practices, and values from parents to children (Kelley et al., 2021; Unser, 2025), often aimed at preserving traditions across generations (Gemar, 2023). In this transmission process, it is not only religious practices that are passed down, but also ways of conceptualizing religious entities. For instance, Cui et al. (2020) found that children’s confidence in the existence of religious entities was associated with their parents’ religiosity level. Similarly, Saide and Richert (2022) found that children of religious parents tended to hold similar conceptions of God to their parents, whereas in non-religious families, parent-child views of God tended to diverge. We note that an individual’s religious beliefs may also be influenced by other external factors, such as societal attitudes toward religion (Gebauer and Sedikides, 2021), hardship in life, or their peer group (Krauss et al., 2012; Peres et al., 2007; Storm, 2017).

Taken together, research highlights the importance of cultural and family contexts in shaping the socialization of scientific and religious beliefs. However, there remains limited cross-cultural understanding of how parents perceive their role in shaping children’s scientific and religious beliefs, or how they respond when their children hold dissenting beliefs. Given that such perceptions are likely to shape parenting practices, this represents an important gap in understanding how scientific and religious beliefs are transmitted across generations. To the best of our knowledge, there is only one published study to date (McLoughlin et al., 2021) that has directly investigated parents’ perceptions of their influence on their children’s scientific and religious beliefs across three diverse cultural groups. McLoughlin et al. (2021) explored whether parents in the U.S., China, and Iran believed they were influential in their children’s scientific and religious views, and whether they were the only source of influence. Parents also commented on how they would approach dissenting – that is, conflicting or deviating – beliefs in their children. The authors broadly categorized the responses as directive, where parents attempted to direct or change their children’s views (e.g., “I would explain to him that he is wrong about this”), and supportive, where parents supported and accepted their children’s different views (e.g., “He is his own person, and his views are his own to form”). This study found that parents in the U.S. and Iran endorsed that they equally influenced their children’s scientific and religious views, while parents in China reported that they influenced their children’s scientific beliefs more than their religious beliefs. Iranian and Chinese parents did not mention other sources of influence for science and religion, whereas American parents included other influences for religion but not for science. When considering their reactions to their children’s scientific/religious views differing from their own, parents in Iran were more likely to express a directive stance, whereas parents in China and the U.S. were more likely to express a supportive stance. These differences suggest that cultural context shapes how parents position themselves as transmitters of scientific and religious beliefs, and how they respond when children hold divergent beliefs. The observed differences in parenting attitudes across cultural groups may reflect variation in the relative valuation of religion and science, dominant religious traditions, and norms surrounding authority and collectivism.

We adapted McLoughlin et al.’s (2021) questions by asking Australian and Malaysian adults to retrospectively consider how much they thought their parents had influenced their current scientific and religious beliefs. Participants also described how their parents influenced their beliefs (if applicable), and how their parents approached situations when they held dissenting scientific/religious beliefs. We focused on such considerations in Australia and Malaysia because they represented contrasting religious landscapes and sociocultural attitudes toward science and religion. Australia is a largely secular nation where Christianity is the predominant religion, and no religion is officially established, imposed, or prohibited (Australian Bureau of Statistics, 2022; Barker, 2018). Malaysia is a religiously diverse society, with Islam, Christianity, Buddhism, and Hinduism as major religious affiliations, although Islam is recognized as the religion of the Federation (Kamarzaman et al., 2024). Malaysia and Iran represent countries with predominantly Islamic religious populations (Hossain, 2013; Karabenick and Moosa, 2005), whereas the U.S. and Australia are becoming increasingly secular, with Christianity representing the most prominent religion (Australian Bureau of Statistics, 2022; Pew Research Center, 2025; Public Religion Research Institute, 2024). This study extends McLoughlin et al.’s (2021) finding that religious and scientific socialization processes differ cross-culturally by considering how adults retrospectively reflect on their own belief development.

## Methods

### Participants

Our sample was comprised of 297 non-parent participants from Australia (*n* = 180; 127 females, 47 males, 3 non-binary, 3 did not report their gender; *M*_*age*_ = 18.71, *SD* = 3.14, range = 17–39 years) and Malaysia (*n* = 117; 85 females, 16 males, 1 non-binary, 15 did not report; *M*_*age*_ = 25.64, *SD* = 5.91, range = 18–50 years). Australian participants were undergraduate students recruited in their university’s psychology program and academic credits were offered as a rewards. Malaysian participants were recruited through Facebook (a popular local social media platform) and WhatsApp (a convenient platform used for official communications in many settings) and were able to enter a lottery to win one of 20 food vouchers, valued at 50 Malaysian Ringgit (RM50) each. All participants completed the survey online via Qualtrics, administered through the university server. Participants’ basic personal information was collected separately at the end of the survey to ensure they were renumerated for participation, and to prevent multiple entries per participant (student ID and name for Australia; email address and name for Malaysia).

Sixty percent of Australian participants were White (*n* = 108), 35% were Asian (*n* = 63), and 5% identified as another ethnicity (*n* = 9). Seventy-eight percent of Malaysian participants were Malay (*n* = 80), 10% were Indian (*n* = 10), 6% were Chinese (*n* = 6), and 6% identified as another ethnicity (*n* = 6). Almost half of the Australian participants identified as atheist (*n* = 88, 49%), 29% as Christian (*n* = 53; Anglican *n* = 3, 2%; Catholic *n* = 33, 18%; Christian *n* = 10, 5%; Protestant *n* = 7, 4%), 5% as Muslim (*n* = 9), 4% as Hindu (*n* = 8), 4% as Buddhist (*n* = 7), 4% as agnostic (*n* = 6), and 5% as belonging to another religion (*n* = 9). The majority of the Malaysian sample identified as Muslim (*n* = 87, 82%), approximately 6% as Hindu (*n* = 6), 5% as Buddhist (*n* = 5), 4% as atheist (*n* = 4), 3% as Catholic (*n* = 3), and one participant identified as belonging to another religion. In general, participants reported having middle socio-economic status (SES; *M*_*Australia*_ = 5.67, *SD*_*Australia*_ = 1.81, *M*_*Malaysia*_ = 5.34, *SD*_*Malaysia*_ = 1.42), measured using the MacArthur Scale of Subjective Social Status^1^ (Adler et al., 2000).

### Measures

#### Participants’ religiosity

Following Payir et al.’s (2020) procedure, participants’ religiosity level was coded using three questions administered in the online survey. Participants received a score of 1 for each item if they: (1) attended a place of worship more than once a month (“How often do you currently attend a place of worship (e.g., Mosque, Church, Temple)?”); (2) self-identified as religious (“Independently of whether you attend religious services or not, would you say you are (a) a religious person, (b) not a religious person”); and (3) engaged in private worship more than once a week (“How often do you privately worship (e.g., pray)?”). We then added these scores to generate a total Religiosity score, with a possible range of 0–3.

#### Parents’ attitudes and beliefs

The online survey presented participants with open-ended, retrospective questions [adapted from McLoughlin et al. (2021)] about their parents’ attitudes toward scientific and religious beliefs in the context of parenting. Participants were asked whether their parents influenced their scientific and religious views (e.g., “Do/did your parents’ views about science/religion influence your scientific/religious views? If so, how?”), and how their parents would react if they held divergent beliefs to themselves (e.g., “How would your parents respond if you developed different scientific/religious views from them?”).

### Coding

We followed McLoughlin et al.’s (2021) coding schemes and coded participants’ open-ended responses dichotomously for the following.

#### Parental influence

Participants’ responses were coded as *affirmation* when they were affirmative about their parent(s) influencing their beliefs (e.g., “Yes. My dad is a huge fan of science and I turned out to be an engineer.”), and *denial* in the opposite case (e.g., “No my parents’ views about science do not influence my scientific views. I gained a better understanding at school.”). Additionally, responses were coded as *ambiguous* when direct parental influence was unclear (e.g., “Somewhat. They encouraged logical thinking.”).

#### Source of influence

For participants who were affirmative about their parents’ influence, we coded their narration of how their parents had influenced their beliefs (“If so, how?”). We focused on the sources of influence and coded whether participants mentioned their *parent only* (e.g., “Yes, my mom always tell me facts related to science when we see something for example caterpillar, eclipse.”), or *multiple (other) sources* (e.g., “Yes, they educate me with online materials and they did bring me to museums for learning purposes.”). Answers that did not include any information about the sources, or were unclear, were coded as *uninformative* (e.g., “yes, it was not religious at all.”).

### Stance toward dissenting views

The attitudes of participants’ parents toward conflict with their children about beliefs were categorized into two main stances: (a) *supportive* approach and (b) *directive* approach. Responses were coded as *supportive* if the participant relayed that their parent endorsed one or both of these two strategies: (1) collaborative discussions or attitudes to understand and support the participant’s point of view, and (2) willingness to accept the participant’s belief (e.g., “We would discuss it openly to understand both views.”). Responses were coded as *directive* if the participant said their parent presented one or both of these two strategies: (1) the intention to instruct them and redirect their beliefs without explicitly considering the participant’s beliefs, and (2) the rejection of their dissenting beliefs (e.g., “They would probably try to convince me otherwise.”). Responses that did not clearly indicate either of the two stances were coded as *uninformative*.

#### Additional coding: valence of parental influence

Additionally, for participants who affirmed that parents influenced their scientific or religious beliefs, we further coded the directionality of participants’ experience of parental influence on engagement with each belief domain. Responses were coded as positive (*p*) when parental influence was described as increasing exposure to, interest in, or endorsement of scientific (e.g., “Yes completely. My father was a science teacher and he has helped shaped my love for science and understanding of the world.”) or religious beliefs (e.g., “Yes. They lead by example. By practicing religion in every inch of their life”). Responses were coded as negative (*n*) when parental influence was described as decreasing exposure to, discouraging, or limiting endorsement of scientific (e.g., “Yes. My parents are religious so I wasn’t taught scientific views.”) or religious beliefs (e.g., “Yes. Their blind faith in religion influenced me to view it very skeptically as I saw their faith as detrimental”). Responses were coded as ambiguous (*a*) when the valence of influence was unclear or neutral (e.g., “Yes, they gave me perspective”).

#### Inter-rater reliability

Non-English (Malaysian) responses were first independently translated by a native speaker and the fourth author (also a native speaker). They then cross-checked their translations and agreed on a final version together; this ensured that cultural and psychological nuances were preserved in translations. All data were independently coded by two research assistants who were not familiar with the study. Level of coders’ agreement varied across instances (Parental Influence, κ = 0.94; Source, κ = 0.78; and Dissent, κ = 0.70 for Science; Parental Influence, κ = 0.92; Source, κ = 0.80; and Dissent, κ = 0.92 for Religion), but was generally acceptable. Disagreements were resolved via discussion between the coders and the first author.

## Results

We analyzed our data using Generalized Linear Mixed Models, with binomial logistic regressions using the lme4 package in R (Bates et al., 2015). Preliminary analysis focusing on gender, age, and SES indicated no main effects and so these were not included in subsequent analyses. In all model comparisons, Country (Australia vs. Malaysia), Domain (Science vs. Religion), and Religiosity were entered as fixed effects and Participants was entered as a random intercept to account for within-subject variation across measures. Models with all two-way interactions improved model fit and those interactions were included. See Table 1 for frequencies of observed data across the three measures.

**TABLE 1:** Proportion of outcome variables for malaysian and australian adults across domains.

Outcome variables	Malaysia science		Malaysia religion		Australia science		Australia religion	
	*n*	%	*n*	%	*n*	%	*n*	%
Parental influence								
Affirmation	53	56.38	82	85.42	90	53.25	106	65.03
Denial	41	43.62	14	14.58	79	46.75	57	34.97
Source								
Parent-only	33	70.22	55	75.34	75	75.00	49	44.95
Multiple	14	29.78	18	24.66	25	25.00	60	55.05
Approach to Dissents								
Directive	14	20.90	44	65.67	23	18.85	34	27.79
Supportive	53	79.10	23	34.33	99	81.15	88	72.31

### Participant religiosity

Religiosity scores (range of 0–3), derived from the three-item Religiosity index, were compared between Australian and Malaysian participants using an independent samples *t*-test. Australian participants reported significantly lower levels of Religiosity (*M* = 0.51, *SD* = 0.89) than Malaysian participants (*M* = 1.66, *SD* = 0.91), *t*(216.25) = −10.35, *p* < 0.001.

### Parental influence

**FIGURE 1:**
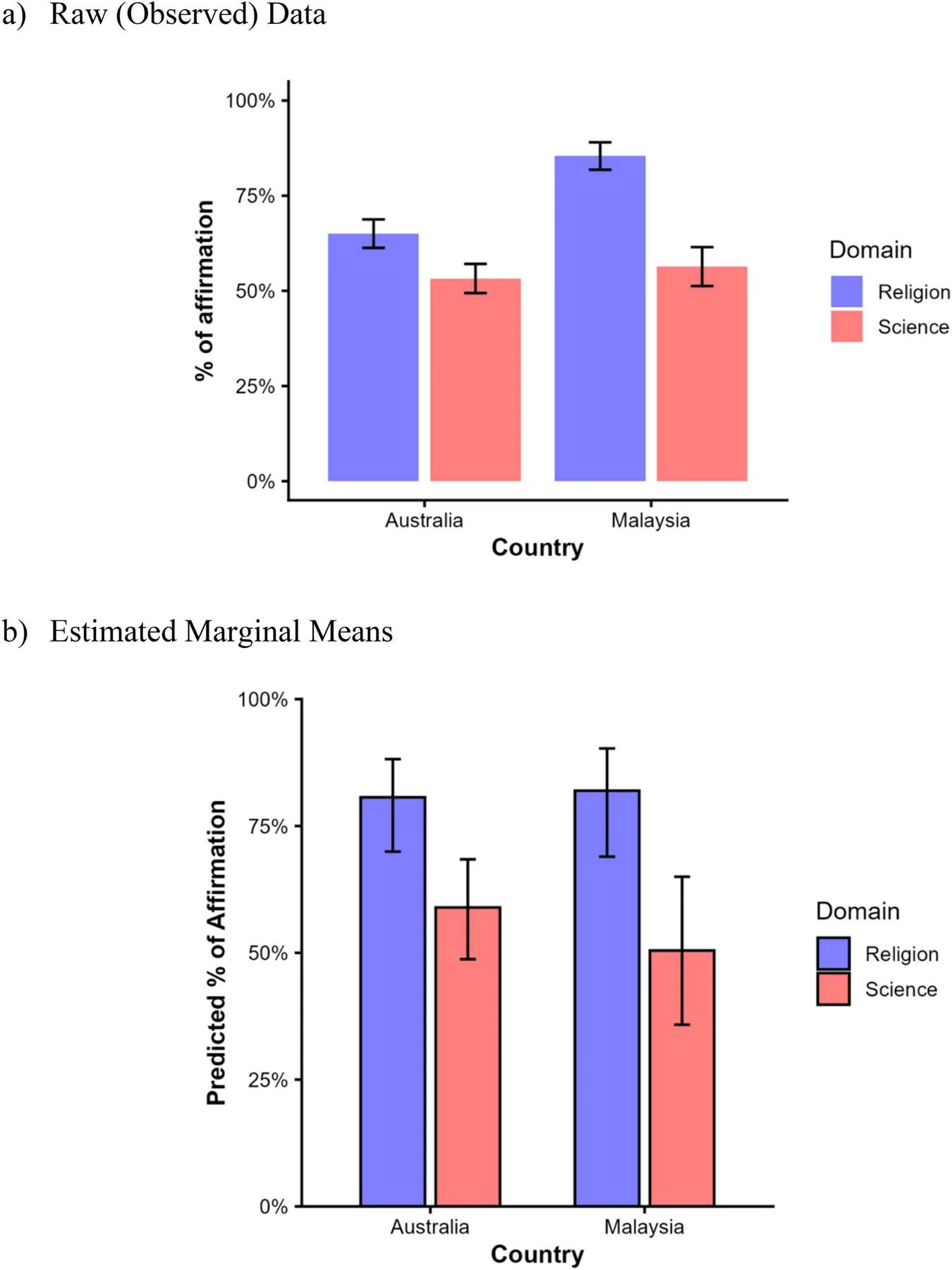
Observed and predicted percentage of affirmation of parental influence in Australia and Malaysia according to domain. **(a)** Raw (observed) data. **(b)** Estimated marginal means. Error bars represent standard errors. The final model did not reveal a significant Country × Domain interaction.

Over half of the participants from Malaysia (56.38%) and Australia (53.25%) affirmed their parents’ influence on their scientific beliefs. Similarly, both Malaysian and Australian participants affirmed the influence of their parents on their religious views, with a larger percentage in Malaysia (85.42%) than Australia (65.03%; see Figure 1a).

This analysis focused on the effect of Country, Domain, and Religiosity on whether participants affirmed or denied parental influence on their beliefs. The final model revealed no significant effect of Domain, *b* = 0.08, *SE* = 0.70, *p* = 0.910, and no significant effect of Country, *b* = 0.16, *SE* = 0.51, *p* = 0.753 (Figure 1b). In general, participants from both countries were equivalently likely to endorse the influence of their parents on their own beliefs for both domains. The Domain × Country interaction was not significant, *b* = −0.43, *SE* = 0.58, *p* = 0.455.

**FIGURE 2:**
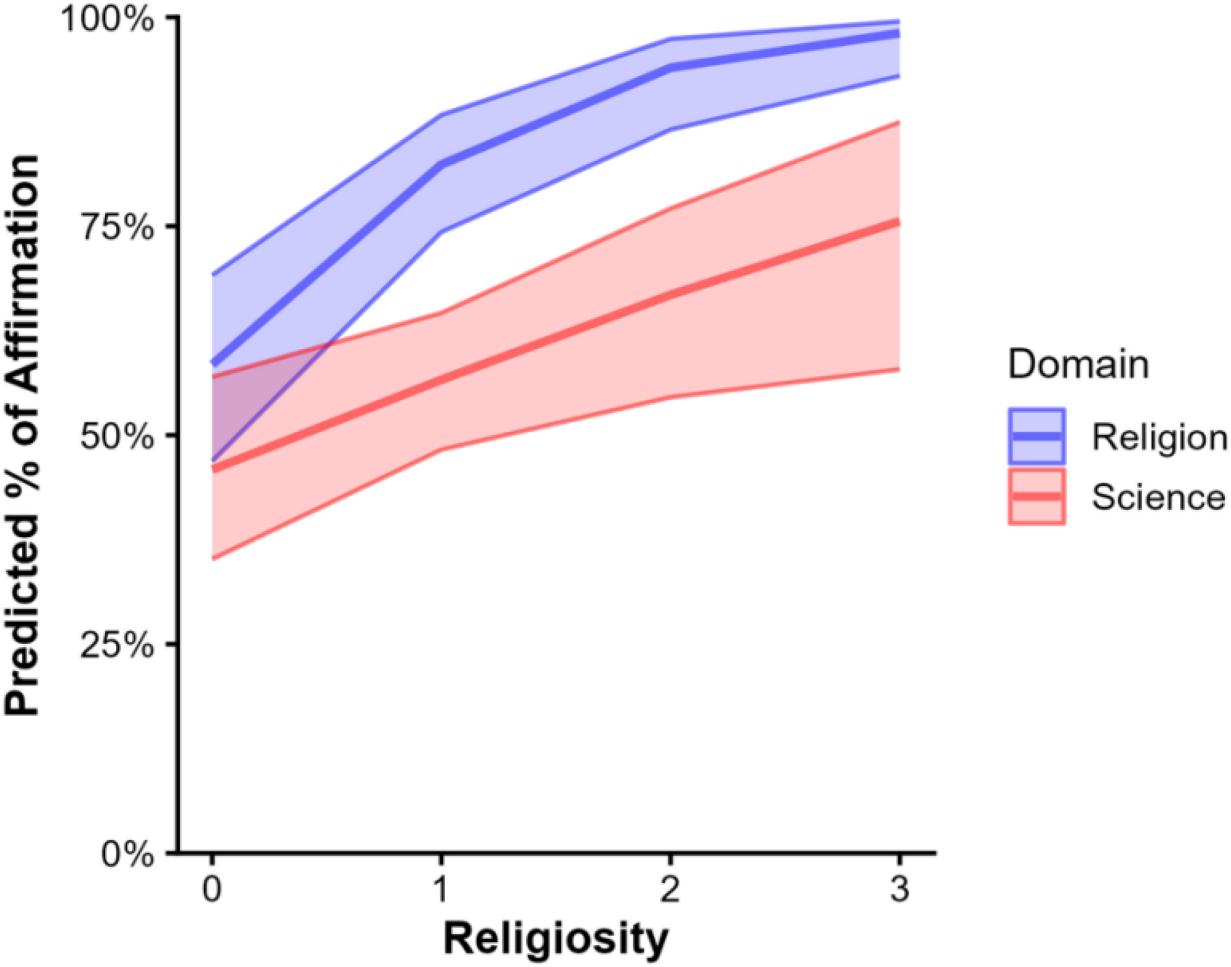
Predicted percentage of affirmation of parental influence (averaged across countries) on religion and science across religiosity levels 0–3.

The model revealed a significant main effect of Religiosity, *b* = 1.31, *SE* = 0.52, *p* = 0.011. The more religious an individual was, the more likely they were to report that their parents influenced their beliefs. Notably, this effect was stronger for religion than science, as indicated by a significant Domain × Religiosity interaction, *b* = −0.77, *SE* = 0.30, *p* = 0.009 (Figure 2). For both samples, increased religiosity was associated with a greater likelihood of affirming parental influence on religious beliefs (Australia, *b* = 1.23, *SE* = 0.30, 95% CI [0.65, 1.82], *p* < 0.001; Malaysia, *b* = 1.15, *SE* = 0.30, 95% CI [0.55, 1.75], *p* < 0.001); this trend was weaker for scientific beliefs (Australia, *b* = 0.46, *SE* = 0.21, 95% CI [0.05, 0.88], *p* = 0.029; Malaysia, *b* = 0.38, *SE* = 0.26, 95% CI [−0.12, 0.88], *p* = 0.137). There was no significant Country × Religiosity interaction, *b* = −0.08, *SE* = 0.30, *p* = 0.788.

#### Valence of parental influence affirmation

Positive science affirmations accounted for more than half of responses, whereas negative science affirmations were rare in both Australian participants (positive = 65, negative = 6, ambiguous = 23) and Malaysian participants (positive = 36, negative = 1, ambiguous = 19). Positive religious affirmations also accounted for more than half of responses; however, negative religious affirmations were more substantial among Australian participants (positive = 68, negative = 23, ambiguous = 18) compared to Malaysian participants (positive = 59, negative = 4, ambiguous = 26).

**FIGURE 3:**
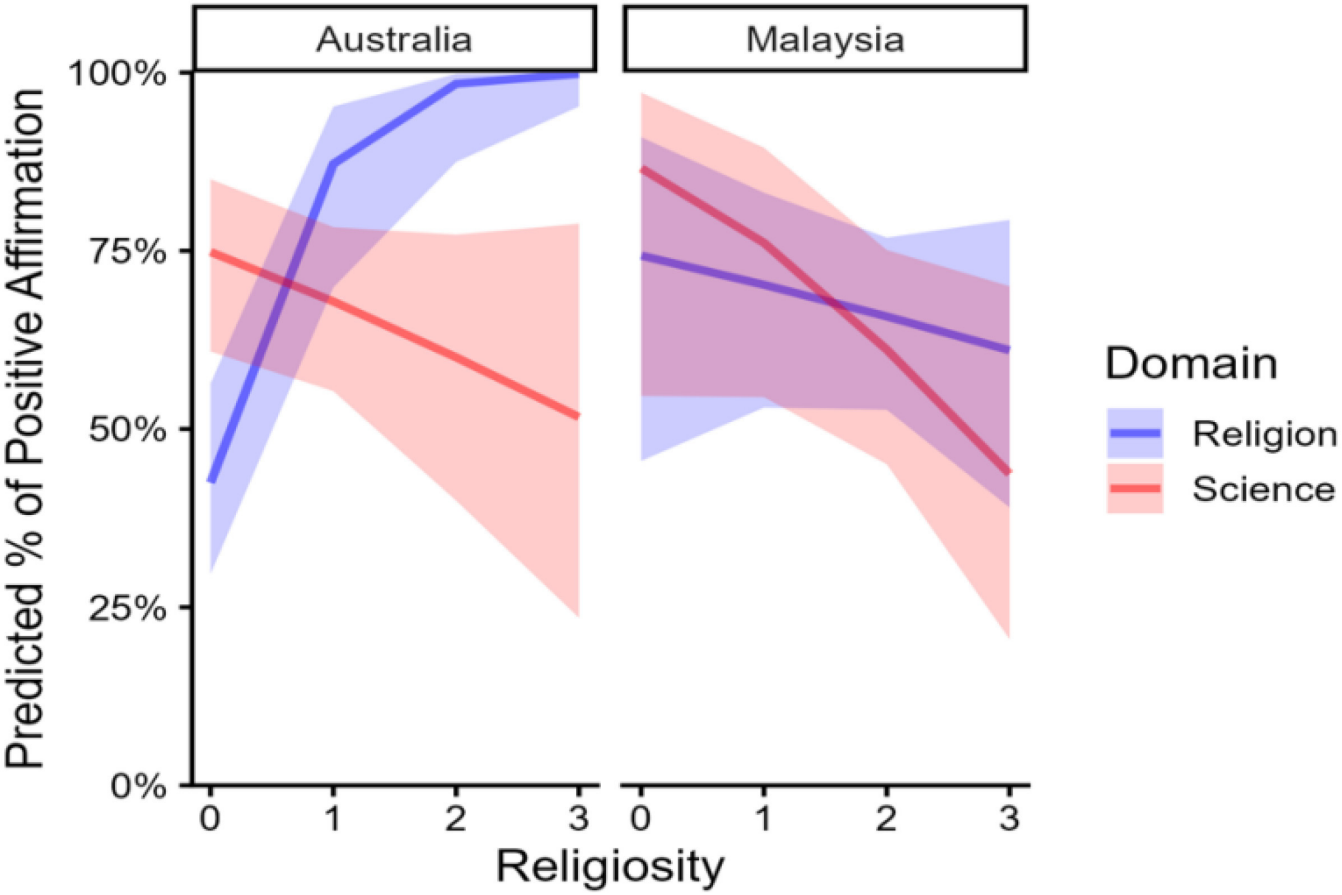
Predicted percentage of positive affirmation on religion and science in Australia and Malaysia across religiosity levels 0–3. We found a significant Religiosity × Domain × Country interaction. The relation between Religiosity and Science was not significant for either Country. The relation between Religiosity and Religion was significant for Australia only.

We dummy-coded the valence of responses (Positive = 1; Negative and Ambiguous = 0) and conducted the same analysis focusing on the effect of Country, Domain, and Religiosity on whether participants viewed either belief positively under their parents’ influence. The three-way interaction term was included in the model as it provided a better fit than the two-way interaction model. The two-way interactions indicated that greater religiosity was associated with higher levels of positive affirmation in the religion domain (Religiosity × Domain, *b* = −2.56, *SE* = 0.67, *p* < 0.001) and among Australian participants (Religiosity × Country, *b* = −2.43, *SE* = 0.69, *p* < 0.001). The significant three-way interaction further indicated that the interaction between Religiosity and Domain was stronger in Australia than in Malaysia (*b* = 2.06, *SE* = 0.83, *p* = 0.01; Figure 3). The model revealed that greater Religiosity was associated with higher levels of positive affirmation in Religion in Australian adults (*b* = 2.22, *SE* = 0.61, *p* < 0.001), but not in Malaysian adults (*b* = −0.71, *SE* = 0.42, *p* = 0.089). Interestingly, the association between Religiosity and positive affirmation in Science was not significant in either Australian adults (*b* = −0.34, *SE* = 0.26, *p* = 0.186) or Malaysian adults (*b* = −0.71, SE = 0.42, *p* = 0.089).

### Source of influence

**FIGURE 4:**
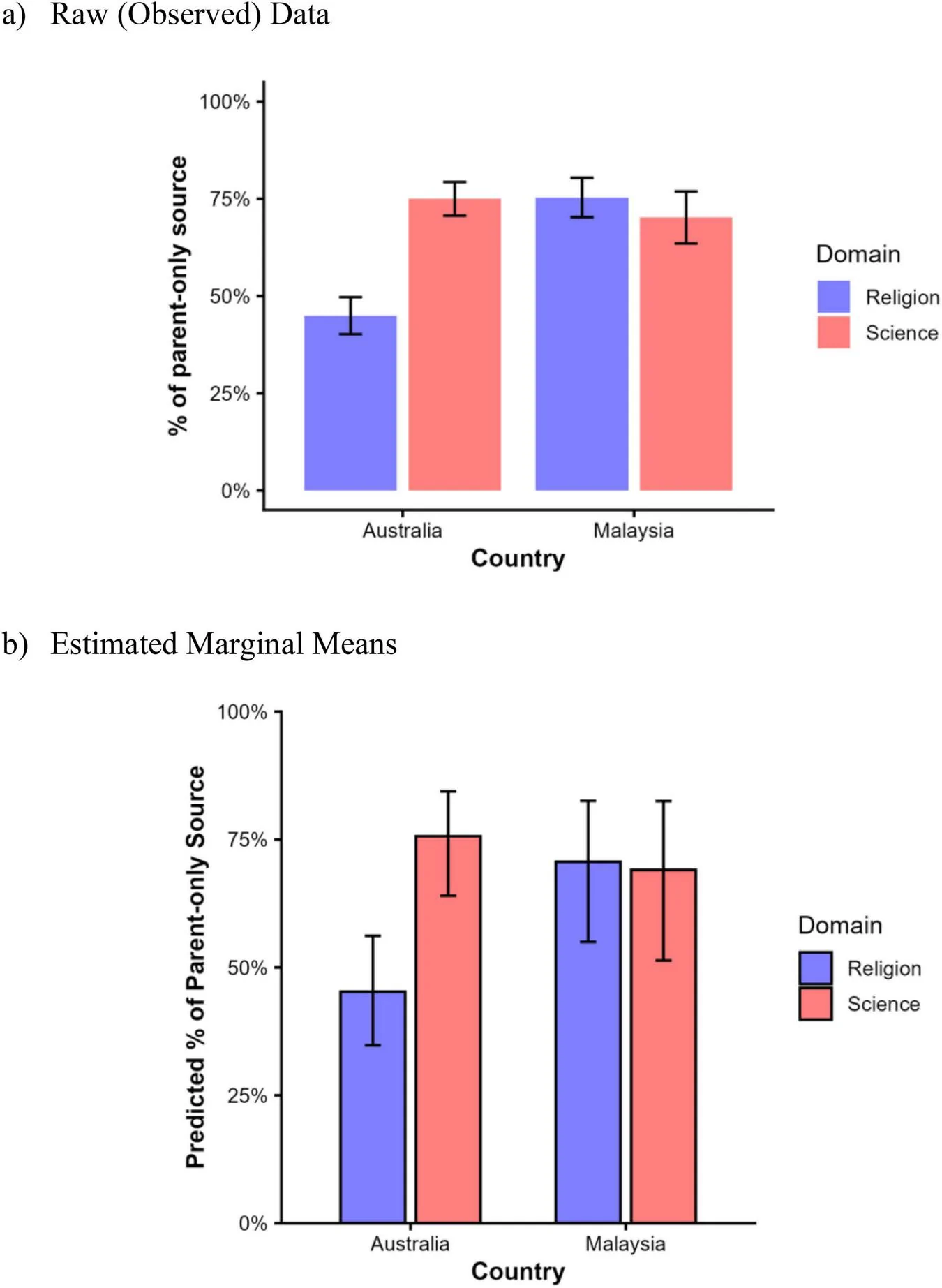
Observed and predicted percentages of parent-only source responses in Australia and Malaysia according to domain. **(a)** Raw (observed) data. **(b)** Estimated marginal means. Error bars represent standard errors. The final model revealed a significant Country × Domain interaction.

Approximately three-quarters of Malaysian participants only mentioned parents as their source of influence for either their scientific or religious beliefs (70.21% for science; 75.34% for religion). This was similar for Australians for their scientific views (75.00%), whereas nearly half of the sample (44.95%) mentioned multiple other sources when talking about influences on their religious views (see Figure 4a).

This analysis focused on the effect of Country, Domain, and Religiosity on Source of Influence (parent-only vs. multiple). The final model found no significant main effects of Country, *b* = 0.62, *SE* = 0.57, *p* = 0.277, and Religiosity, *b* = 0.11, *SE* = 0.20, *p* = 0.588. Participants’ Religiosity level did not influence their judgments of whether they had acquired their beliefs from parent-only or multiple sources. However, there was a main effect of Domain, *b* = 1.64, *SE* = 0.40, *p* < 0.001. Overall, participants were more likely to mention parent-only sources for science, *M* = 0.73, *SE* = 0.05, 95% CI [0.62, 0.81], *p* = 0.083, as compared to religion, *M* = 0.58, *SE* = 0.05, 95% CI [0.49, 0.68], *p* < 0.001. This effect was particularly driven by the Australian sample, as indicated by a significant Domain × Country interaction, *b* = −1.40, *SE* = 0.61, *p* = 0.022 (Figure 4b). *Post hoc* comparisons revealed that Malaysian participants’ likelihood of mentioning parent-only sources was equivalent across domains, *M*_*Science*_ = 0.69, *SE_*Science*_* = 0.08, 95% CI [0.51, 0.83], *p* = 0.036; *M*_*Religion*_ = 0.71, *SE_*Religion*_* = 0.07, 95% CI [0.55, 0.83], *p* = 0.011. By contrast, Australian participants were more likely to identify parents as their only source of influence for science, *M* = 0.76, *SE* = 0.05, 95% CI [0.64, 0.84], *p* < 0.001, than they were for religion, *M* = 0.45, *SE* = 0.06, 95% CI [0.35, 0.56], *p* = 0.394. The interactions of Country × Religiosity, *b* = 0.44, *SE* = 0.30, *p* = 0.147, and Domain × Religiosity, *b* = −0.31, *SE* = 0.27, *p* = 0.244, were not significant.

### Stance toward dissenting views

**FIGURE 5:**
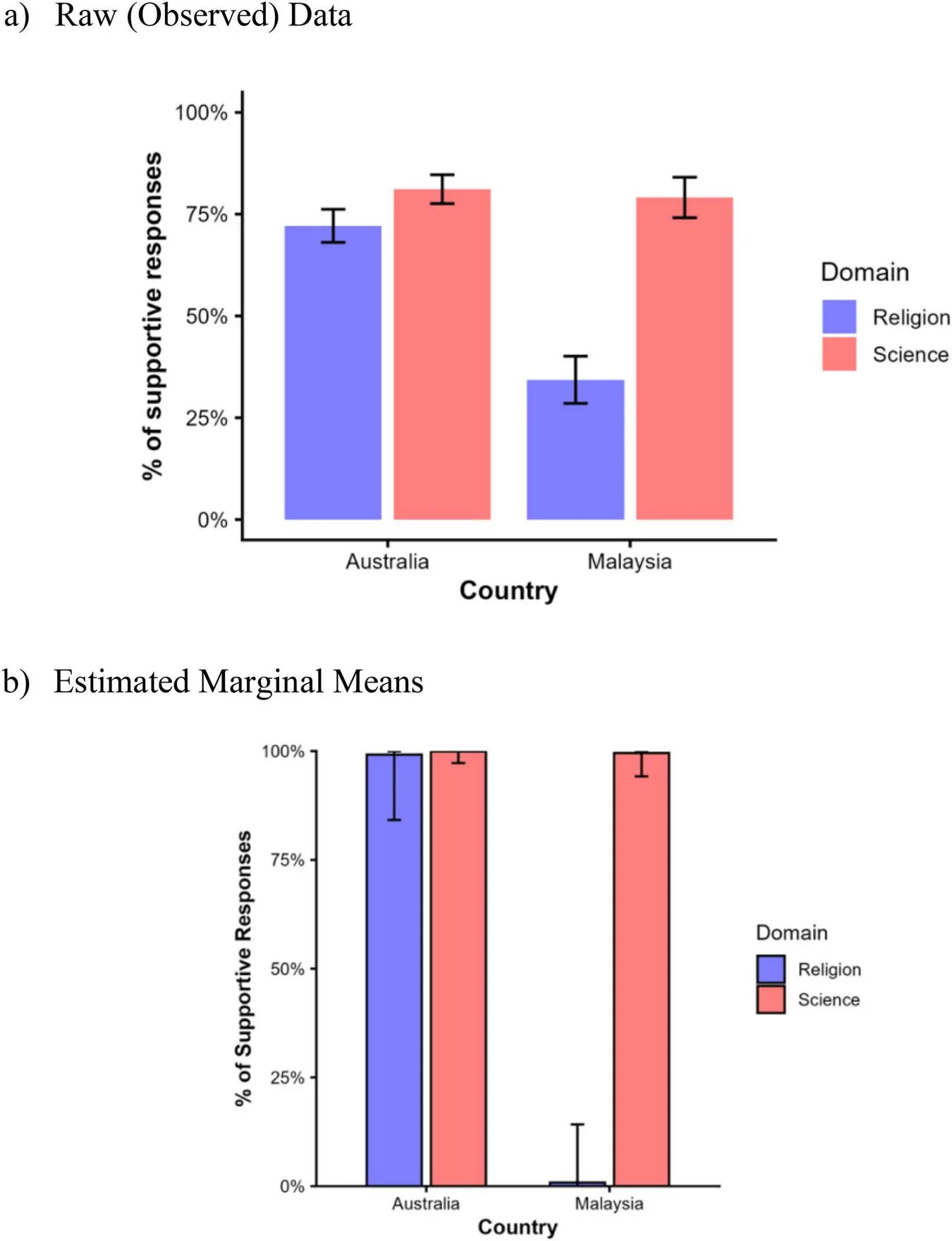
Observed and predicted percentages of supportive responses in Australia and Malaysia according to domain. **(a)** Raw (observed) data. **(b)** Estimated marginal means. Error bars represent standard errors. The final model revealed a significant Country × Domain interaction.

Only 34.33% (*n* = 23; *n* = 44, 65.67% directive) of Malaysian participants recalled their parent using a supportive approach in matters of religion, whereas 79.10% (*n* = 53; *n* = 14, 20.90% directive) reported a supportive approach for science. The approach reported by Australian participants was consistently supportive across both domains, with 81.15% (*n* = 99; *n* = 23, 18.85% directive) of respondents reporting a supportive stance in science and 72.13% (*n* = 88; *n* = 34, 27.87% directive) in religion (see Figure 5a).

This analysis focused on the effect of Country, Domain, and Religiosity on Stance Toward Dissenting Views (directive vs. supportive). The final model revealed a significant effect of Country, *b* = −11.79, *SE* = 2.73, *p* < 0.001, with Australian participants almost twice as likely to report supportive stances, *M* = 1.00, *SE* = 0.004, 95% CI [0.94, 1.00], *p* < 0.001, than Malaysian participants, *M* = 0.58, *SE* = 0.25, 95% CI [0.16, 0.91], *p* = 0.752. It also revealed a significant effect of Domain, *b* = 1.54, *SE* = 0.90, *p* = 0.09, suggesting that participants were significantly more likely to mention their parents’ supportive stances in regard to conflicting views for science, *M* = 1.00, *SE* = 0.002, 95% CI [0.97, 1.00], *p* < 0.001, than for religion, *M* = 0.50, *SE* = 0.20, 95% CI [0.18, 0.83], *p* = 0.986. However, this was mainly driven by the Malaysian sample, as indicated by a significant Domain × Country interaction, *b* = 7.71, *SE* = 2.00, *p* < 0.001 (Figure 5b). Australian participants were similarly likely to report that their parents took a supportive approach for both domains, *M*_*Science*_ = 0.99, *SE_*Science*_* = 0.01, 95% CI [0.84, 1.00], *p* < 0.001; *M*_*Religion*_ = 1.00, *SE_*Religion*_* = 0.01, 95% CI [0.84, 1.00], *p* = 0.003. Contrastingly, Malaysian participants’ parents were more likely to employ a supportive approach toward dissenting scientific views, *M* = 1.00, *SE* = 0.01, 95% CI [0.94, 1.00], *p* < 0.001, compared to religious views, *M* = 0.01, *SE* = 0.01, 95% CI [0.0004, 0.14], *p* = 0.002. *Post hoc* cross-country comparisons revealed a significant difference in approaches for religion, *p* = 0.002, but not science, *p* = 0.721.

**FIGURE 6:**
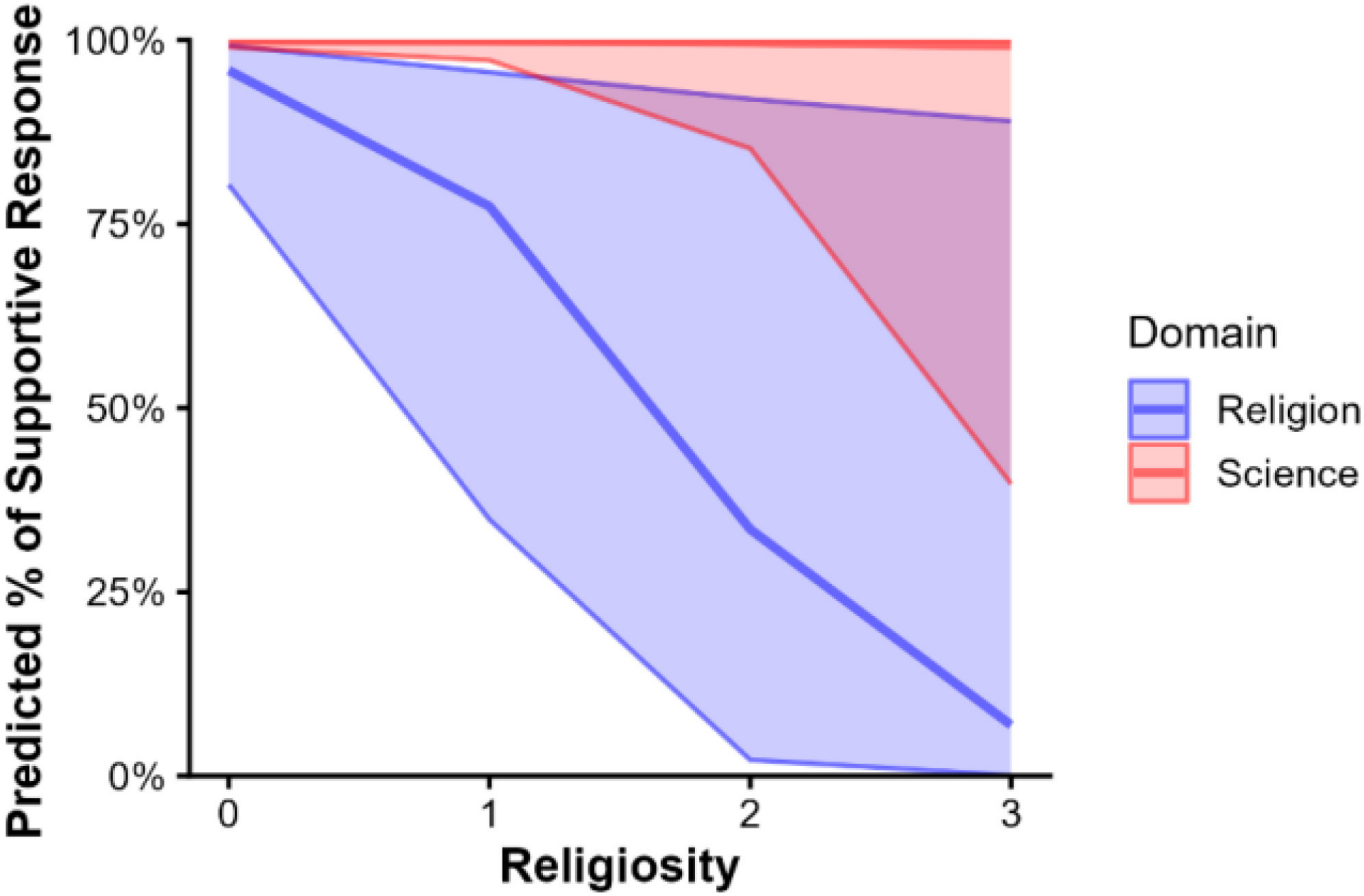
Predicted percentage of supportive response (averaged across countries) according to domain across religiosity levels 0–3.

Additionally, our model also revealed a main effect of Religiosity, *b* = −2.77, *SE* = 1.21, *p* = 0.022. The interactions of Domain × Religiosity, *b* = 1.09, *SE* = 0.68, *p* = 0.110, and Country × Religiosity, *b* = 2.40, *SE* = 1.42, *p* = 0.090, were not significant (Figure 6). Regardless of belief domain and participants’ country, the more religious an adult was, the more likely they were to report their parents taking a directive approach against conflicting views between them.

## Discussion

We adapted a previous method (McLoughlin et al., 2021) to evaluate Australian and Malaysian adults’ perceptions of their parents’ influence on their scientific and religious beliefs. Across countries and domains, over half of the sample endorsed their parents’ influence on their own beliefs, and our statistical model suggested that the likelihood of doing so did not differ across domains. Whereas the majority of Malaysian participants reported their parents as the sole source of influence on both religious and scientific beliefs, Australian participants tended to identify their parents as the sole influence for scientific beliefs, but were relatively less likely to do so for religious beliefs. That is, in addition to parental influence, over half of the Australian sample recalled that their religious views were also influenced by other sources, such as friends and religious organizations. These findings are in line with McLoughlin et al.’s (2021) conclusions; cross-cultural differences exist in individuals’ affirmation of parental influence on beliefs, and the mention of multiple sources of influence. Specifically, our Australian pattern was similar to that observed in the U.S., and our Malaysian pattern was similar with that observed in Iran previously.

These observed patterns in reported sources of influence may partly reflect the framing of the response measure, which asked participants to indicate whether their parents had influenced their beliefs. As such, the responses may have been shaped by a priming effect, in which participants were prompted to consider parental influence specifically. The open-ended nature of the response nonetheless suggested that parents remain a significant source in participants’ acquisition of beliefs, as parental influence was the only source reported in many cases, especially for science beliefs. This is notable given that participants would have acquired a substantial amount of scientific knowledge from schools, books, and media; however, when prompted to reflect on their scientific beliefs, they focused on parents and mostly described their experiences positively, regardless of religiosity and country. This reflects that our question primarily captured retrospective parental influence on participants’ attitudes toward science, rather than the broader sources contributing to their scientific knowledge (McPhetres and Zuckerman, 2018).

The majority of the Australian participants did not identify with a religion, and Australian statistics suggest that many of their parents were likely Christians (Australian Bureau of Statistics, 2022). In such cases where participants do not practice the same religion as their parents, their religious views may have been shaped by a broader set of sources beyond their parents. What remains unclear is how the broad term of “religious views” may also encompass worldviews that do not necessarily align with mainstream religious teachings, including cases where parental influence may have been experienced negatively. For example, an individual may have been influenced by a Christian parent in a negative way, while also being exposed to other influences, ultimately adopting a non-Christian worldview, highlighting the complex integration of multiple influences. Our results provide a preliminary indication of this complexity, with some participants describing parental influence as “negative,” leading to reduced engagement in and endorsement of religious beliefs. This was more prominent in our Australian sample (noting that individuals who were more religious reported positive views about their parents’ influence), whereas parental influence on religion in our Malaysian sample was largely received positively. Future research could explore how such parent and non-parental influences are integrated in the development of religious and broader worldviews.

As in McLoughlin et al. (2021), we also examined adults’ reflections regarding how their parents approached dissenting views between parent and child. Australian parents tended to be supportive for both domains. Such propensity for supportive attitudes in general may reflect cultural norms and attitudes toward parenting (Lansford, 2022). Although the Malaysian adults indicated that their parents were similarly open to accepting dissenting scientific views, they reported that their parents were more likely to hold a directive stance when their children expressed divergent religious views. This difference suggests that the parental directive stance may be uniquely attributed to religious topics, rather than an authoritarian parenting style, which is common among Malaysian households (Mofrad and Uba, 2014). Indeed, it appears more likely that parents utilized a less supportive form of communication to prevent potential compromise, possibly reflecting doctrinal influences, such as the treatment of apostasy and social virtues in Islam (Adil, 2007). Additionally, this inclination could be influenced by considerations of a shared national and ethnic identity, given that the sample predominantly comprised Malay individuals, whose cultural identity is closely intertwined with Islam (Hossain, 2013; Nath, 2015). In contrast, science is constantly changing as new data surfaces and theories are updated (Hoy and Adams, 2016). This preliminary nature of scientific knowledge may result in more supportive approaches to divergent views, given that disagreement does not necessarily threaten identity or social cohesion in the same way as religion. Thus, when discussing science topics, Malaysian parents may prioritize helping their children develop accurate understandings of the world and themselves, rather than creating a group identity. We however acknowledge that participants’ responses regarding parent-child scientific disagreement were more difficult to classify into binary categories (supportive vs. directive) than those regarding religious dissent (as indicated by relatively lower level of inter-coder agreement). This may reflect the inherently complex nature of parental responses to dissenting scientific beliefs, and suggests that participants’ interpretations of such responses are similarly multifaceted. Overall, the different approaches toward dissenting scientific and religious beliefs do not suggest that parents view one as more important than the other, but rather highlight that parents may view science and religion as serving different functions in their children’s development, with science supporting understandings of how the world works and religion contributing to group identity (Braswell et al., 2012).

Intriguingly, Malaysian participants tended to conflate science and religion when describing their parents’ scientific influence. For example, some responses reflected the view that scientific understanding supports religious beliefs (e.g., “understanding science helps to understand the creator of all the things in the world.”), and some adults simultaneously drew on both domains as sources of authority and explanation (e.g., “based on the latest scientific evidence and religious text.”). This subtle observation of science and religion co-existence and non-conflict is in line with previous evidence from Iran, another Muslim-majority country (Davoodi et al., 2018; Payir et al., 2020). We did not further investigate this observation because the number of cases was inadequate for meaningful analysis and interpretation. Future research could explore whether Malaysians have a particular tendency to integrate science and religion, and whether this conflation occurs in other contexts, such as in other Muslim-majority countries or highly religious societies more broadly (Thigpen et al., 2020).

In our study, religious individuals were more likely to report that their parents influenced their religious beliefs, but were slightly less likely to affirm that their parents influenced their scientific beliefs. In general, religious individuals were also more likely to report that their parents were more conservative and less open to accepting divergent scientific and religious views. Their religiosity did not influence their judgments of whether their beliefs were solely influenced by parents or by multiple other sources. This was consistent across Australia and Malaysia, suggesting that religiosity may influence belief transmission in similar, universal ways across cultural and religious backgrounds.

We note that this study is limited by differences in recruitment methods between Australian and Malaysian samples. The Australian sample consisted of undergraduate psychology students, whereas the Malaysian sample was recruited from the broader public, resulting in an older average age and a more prominent gender imbalance in the Malaysian sample relative to the Australian sample. However, the mean socio-economic status scores of the two samples were similar. Importantly, analyses indicated no main effects of age, gender or socio-economic status, suggesting these demographic differences did not systematically influence the results. Nevertheless, one might still argue that undergraduate students hold more immediate retrospective viewpoints on parental influence compared to older working adults due to a recency effect (“reminiscence bump” in autobiography memory, see Janssen et al., 2005). Alternatively, older adults, having achieved greater structural and financial independence may perceive parental influence differently. Having a closer look at the general difference between our Australian (younger) and Malaysian (slightly older) samples rules out such age-related alternative explanation. The key differences between two samples lay in the religion domain only. Specifically, Australians (younger adults) reported learning about religion from multiple sources, indicated that their parents were general supportive when their religious views differed from those of their parents, and religiosity was associated with parental influence in Australia but not in Malaysia. It is more logical to thus suggest that either religion or culture plays a role where future research could also focus on further investigating potential intergenerational differences in the level and kind of parental influences.

Our participants were also non-parents providing retrospective judgments of their parents’ influences, whereas previous study (McLoughlin et al., 2021) involved parents reporting their own experiences with their children. This difference in perspective is important to acknowledge, as studies have found a difference between the mothers’ and children’s perceptions of parenting styles; mothers held more favorable views of their parenting than did their children (*generational stakes hypothesis*, Cho et al., 2020). However, we did not observe this in our study given that participants’ reports of parental influence were similar to those observed previously (McLoughlin et al., 2021). Because of its retrospective nature, this study did not capture the specific mechanisms through which beliefs are transmitted, such as whether parents model beliefs through behavior (e.g., enacting religious values or demonstrating scientific reasoning) or engage in more explicit forms of instruction. Future research could therefore examine how different transmission strategies shape children’s beliefs. In addition, for individuals who identified multiple sources of influence, it may be valuable to explore whether this reflects greater epistemic openness or an awareness of one’s own knowledge limitations; this could provide insight into individual differences in how people develop, integrate, and update beliefs. Furthermore, belief transmission is unlikely to be unidirectional; children may also influence their parents’ beliefs, suggesting a bidirectional process that unfolds over time (Gervais et al., 2011; Hendricks et al., 2024; Hwang et al., 2017; Lanman, 2012; Liu and Kaida, 2024). These directions for future research highlight the complexity of belief transmission, consistent with communication frameworks such as the Shannon–Weaver model (Graf, 2024; Shannon and Weaver, 1949), which emphasizes that messages are not simply transmitted but actively interpreted.

Taken together, our findings extend current understanding of cross-cultural variation in scientific and religious belief transmission and highlight the need for continued research focusing on its multidimensional and dynamic nature. The current results validate that parents play a vital role in passing down and maintaining beliefs, further suggesting that variation in parental influence across science and religion may be shaped more by religious or cultural context than by geographical location. Ongoing investigations on this topic with other religious groups promise to afford important insights into the interplay between parental roles, cultural/religious contexts, and the transmission of scientific and religious beliefs across generations.

## Data Availability

The raw data supporting the conclusions of this article will be made available by the authors, without undue reservation.

## Footnotes

1 The MacArthur Scale of Subjective Social Status was used to measure participants’ perceived SES (Adler et al., 2000). Participants were asked to visualize people in their community on a ladder from the least educated and affluent at the bottom, represented by the number 0, to the most educated and rich at the top, represented by the number 10. Participants then indicated where they would place themselves in this range.
